# Metabolic Changes by Wine Flor-Yeasts with Gluconic Acid as the Sole Carbon Source

**DOI:** 10.3390/metabo11030150

**Published:** 2021-03-06

**Authors:** Minami Ogawa, Jaime Moreno-García, Lucy C. M. Joseph, Juan C. Mauricio, Juan Moreno, Teresa García-Martínez

**Affiliations:** 1 Department of Agricultural Chemistry, Edaphology and Microbiology, Severo Ochoa (C6) Building, Agrifood Campus of International Excellence ceiA3, University of Córdoba, 14014 Córdoba, Spain; amigawa@ucdavis.edu (M.O.); mi1gamaj@uco.es (J.C.M.); qe1movij@uco.es (J.M.); mi2gamam@uco.es (T.G.-M.); 2 Department of Food Science and Technology, 392 Old Davis Rd, University of California, Davis, CA 95616, USA; 3 Department of Viticulture and Enology, 595 Hilgard Lane, University of California, Davis, CA 95616, USA; cmjoseph@ucdavis.edu

**Keywords:** *Saccharomyces cerevisiae*, flor yeast, gluconic acid consumption, metabolomics

## Abstract

Gluconic acid consumption under controlled conditions by a *Saccharomyces cerevisiae* flor yeast was studied in artificial media. Gluconic acid was the sole carbon source and the compounds derived from this metabolism were tracked by endo-metabolomic analysis using a Gas Chromatography-Mass Spectrometry (GC-MSD) coupled methodology. After 6 days, about 30% of gluconic acid (1.5 g/L) had been consumed and 34 endo-metabolites were identified. Metabolomic pathway analysis showed the TCA cycle, glyoxylate-dicarboxylate, glycine-serine-threonine, and glycerolipid metabolic pathway were significantly affected. These results contribute to the knowledge of intracellular metabolomic fluctuations in flor yeasts during gluconic acid uptake, opening possibilities for future experiments to improve their applications to control gluconic acid contents during the production of fermented beverages.

## 1. Introduction

Flor yeasts are strains of *Saccharomyces cerevisiae* that are able to aggregate and form a biofilm on air-liquid interface. These biofilms are called flor or velum and form after the end of alcoholic fermentation, when the assimilable nitrogen and fermentative carbon sources are depleted [1]. The formation of a flor-velum layer on the wine surface, where they have access to dissolved oxygen, allows yeasts to float and maintain and shift from fermentative to oxidative metabolism [2].

The flor yeasts are used commonly in the biological aging processes of Sherry and Sherry-like wines [3] or for yeast immobilization techniques for alcoholic fermentation, where yeast cells are attached to a certain region of space with preservation to their biological activity [4]. They can also act as a biosorption tool to naturally decrease undesired compounds such as ethanol or gluconic acid. Ethanol can be consumed by the flor yeasts once they switch to aerobic metabolism after forming the flor on the liquid surface. This is ideal to treat alcoholic beverages with higher than intended alcohol levels, such as wines due to fermentation of overripe grapes [5]. Along a similar concept, flor yeasts are among specific yeast strains that can metabolize gluconic acid [6,7,8,9,10,11]. Gluconic acid is an easily assimilable carbon source by many microbes but it is not fermentable by yeasts [12]. High concentration of gluconic acid make fermented products microbiologically unstable and alter the sensory properties, causing problems in long term storage [13]. Alcoholic products, such as wine, mead, beer or bioethanol, are at risk of having excess gluconic acid if the initial raw material had substantial rot or bacterial contamination [14,15]. The flor yeasts have already been proven effective in reducing gluconic acid in wines [16] and simultaneously reducing volatile acidity as well as increasing butanoic, isobutanoic, 2-methylbutanoic, and 3-methylbutanoic acid, resulting in a wine with more favorable aroma characteristics [6].

While these past findings have proven the efficiency of flor yeast impact on sensory attributes, the mechanism and associated secondary effects of gluconic acid consumption in flor yeasts are not well understood. This limits improvement of the technique as well as application of these *S. cerevisiae* strains for other fruit-based fermented beverages or bioethanol production, which could benefit from their properties. To gain a more complete view of the changes in flor yeast gluconic acid metabolism, this study concentrated on the endo-metabolomics by reporting untargeted endo-metabolite fluctuations in a synthetic medium with gluconic acid as the sole carbon source under controlled conditions. The data presented in this study can be utilized as a basis of knowledge to understand gluconic acid metabolism and improve gluconic acid consumption in flor yeasts in different fermentation sets.

## 2. Results

### 2.1. Gluconic Acid Consumption and Cell Viability

**Figure 1:**
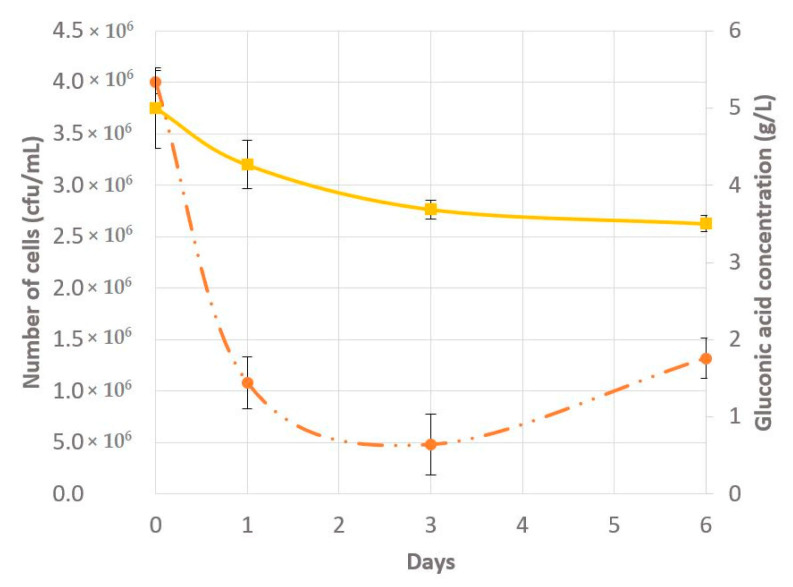
Viability of yeast cells at various time points represented in days of incubation in gluconic acid medium (orange dotted line). Gluconic acid concentration (g/L) in the media at various time points represented in days of incubation in gluconic acid medium (yellow solid line).

The gluconic acid consumption rate showed a gradual decline. At the end of the six days approximately 1.5 g/L or 30% of total gluconic acid had been consumed (Figure 1). The yeast viability dropped in the first three days, in correspondence with an adaptation period for the yeast to the nutrient poor and difficult to metabolize carbon source medium (Figure 1). Typical viability of yeasts in media with non-fermentative carbon sources ranges between 25% and 35% [17]. However, once this adaptation period was established, a viability increase was seen from day 3 to day 6 and would likely increase more, along with gluconic acid consumption, if the experiment was continued.

### 2.2. Endo-Metabolomic Analysis

**Figure 2:**
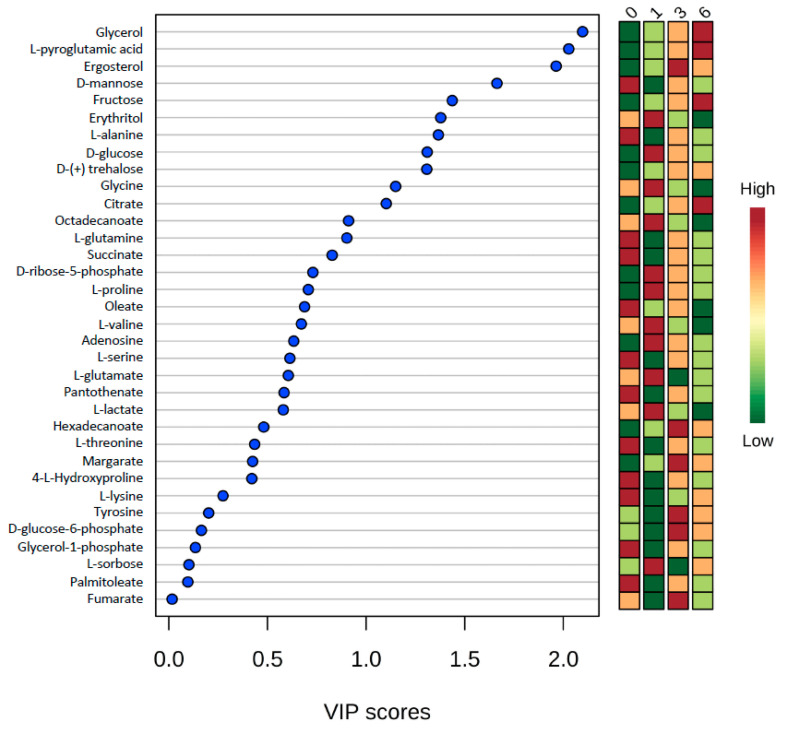
VIP score chart of all metabolites where VIP is Variable Importance in Projection, calculated as the weighted sum of squares of the PLS loadings taking into account the amount of explained Y-variation, in each dimension. The heatmap on the right represents concentration of metabolites at the time points where 0 = day 0, 1= day 1, 3 = day 3, 6 = day 6.

**Figure 3:**
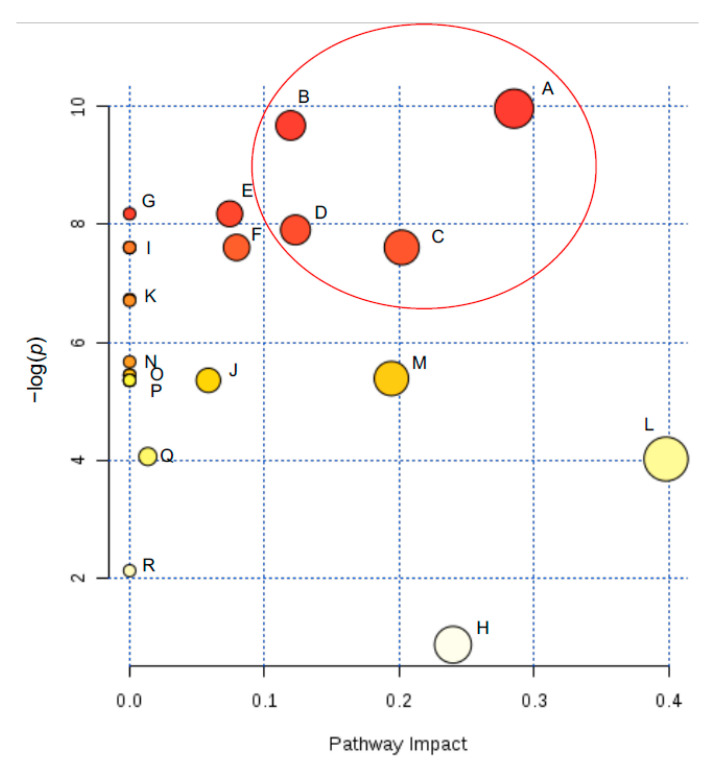
Pathway graph of top 15 VIP scored metabolites according to the *p* values from the pathway enrichment analysis and pathway impact values from the pathway topology analysis. The color of the circle represents the *p*-values (red being higher) and the radius of the circle represents impact (where larger radius is higher impact). The four pathways with highest −log(*p*) and impact value are circled in red. [A] Glycerolipid metabolism, [B] Glyoxylate and dicarboxylate metabolism, [C] Glycine, serine and threonine metabolism, [D] Citrate cycle (TCA cycle), [E] Glutathione metabolism, [F] Methane metabolism, [G] Aminoacyl-tRNA biosynthesis, [H] Pentose phosphate pathway, [I] Cyanoamino acid metabolism, Thiamine metabolism, Porphyrin and chlorophyll metabolism, [J] Sulfur metabolism, [K] Steroid biosynthesis, Amino sugar and nucleotide sugar metabolism, [L] Starch and sucrose metabolism, [M] Alanine, aspartate and glutamate metabolism, [N] Biosynthesis of unsaturated fatty acids, [O] Arginine biosynthesis, Pyrimidine metabolism, Nitrogen metabolism Amino sugar and nucleotide sugar metabolism., [P] Tyrosine metabolism, Propanoate metabolism, Butanoate metabolism, Nicotinate and nicotinamide metabolism, Fructose and mannose metabolism, [Q] Purine metabolism, [R] Galactose metabolism.

**Figure 4:**
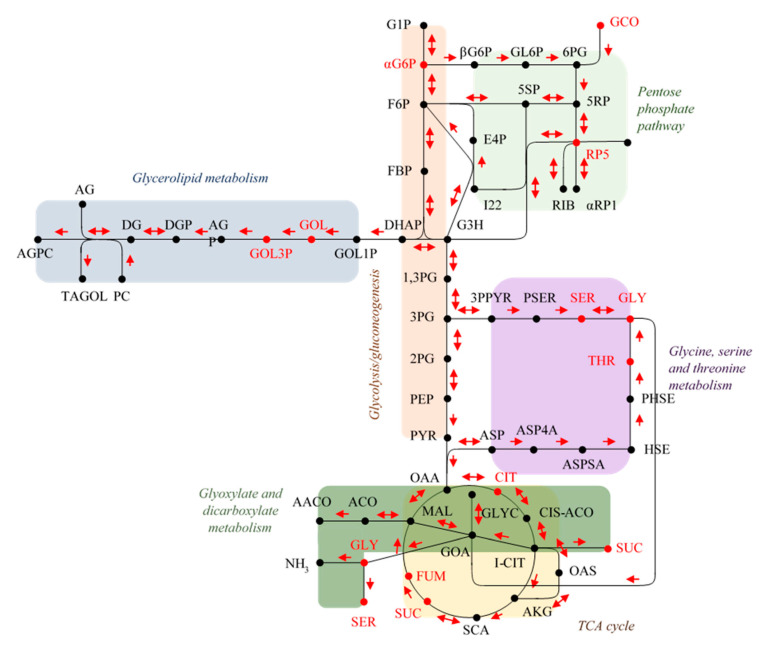
Metabolomic map of flor yeast gluconic acid consumption. Metabolites quantified are represented in red. SGD YeastPathways and KEGG PATHWAY Database—GenomeNet have been used for pathways and gPDB-CCD, SGD, Sigma Aldrich and CHEBI abbreviations have been used for metabolites labelling: 1,3PG: 3-Phospho-d-glyceroyl phosphate; 1,5RP: 5-phospho-alpha-d-ribose 1-diphosphate; 2PG: 2-phosphoglycerate; 3PG: 3-phosphoglycerate; 3PPYR: 3-phosphonooxypyruvate; 5RP: ribulose 5-phosphate; 5SP: xylulose 5-phosphate; 6PG: 6-phospho-d-gluconate; αG6P: alpha-d-Glucose 6-phosphate; αRP1: alpha-d-ribose 1-phosphate; βG6P: beta-d-glucose 6-phosphate; AACO: Acetoacetyl-CoA; ACO: acetyl-CoA; AG: 1-Acylglycerol; AGP: 1-Acyl-sn-glycerol 3-phosphate; AGPC: a 1-acyl-sn-glycero-3-phosphocholine; AKG: 2-oxoglutarate; ASP: l-aspartate; ASP4P: l-aspartyl-4-phosphate; ASPSA: l-aspartyl-4-phosphate; l-aspartate 4-semialdehyde; CIS-ACO: cis-aconitase; CIT: citrate; DG: 1,2-Diacyl-sn-glycerol; DGP: a 1,2-diacyl-sn-glycerol 3-phosphate; DHAP: dihydroxyacetone phosphate; E4P: erythrose 4-phosphate; F6P: fructose 6-phosphate; FBP: fructose 1,6-bisphosphate; FUM: fumarate; G1P: glucose 1-phosphate; G3H: glyceraldehyde 3-phosphate; GCO: gluconic acid; GL6P: d-glucono-1,5-lactone 6-phosphate; GLY: glycine; GLYC: glycolate; GOA: glyoxylate; GOL: glycerol; GOL1P: glycerol 1 phosphate; GOL3P: glycerol 3-phosphate; HSE: l-homoserine; I22: sedoheptulose 7-phosphate; I-CIT: isocitrate; MAL: malate; OAA: oxalacetate; OAS: oxalasuccinate; PC: phosphatidylcholine; PEP: phosphoenolpyruvate; PHSE: O-phospho-l-homoserine; PSER: O-phospho-l-serine; PYR: pyruvate; RIB: d-ribose; RP5: ribose 5-phosphate; SCA: succinyl-CoA; SER: serine; SUC: succinate; TAGOL: triacylglycerol; THR: threonine.

To observe the evolution of intracellular metabolism of yeast cells during the adaptation and gluconic acid consumption period, untargeted metabolomics provided a global overview of metabolites that were depleted, over accumulated, fluctuated over time (low-to-high-to-low or from high-to-low-to-high) or remained stable in their contents at each time point. Fluctuations in the metabolite concentration over time may be due to punctual transformations of metabolites at different physiological stages (i.e., day 0, 1, 2 or 6). 34 total endo-metabolites were determined and plotted in VIP score chart (Figure 2). Quantified metabolites included 12 amino acids, 5 fatty acids, 5 mono- and di-saccharides, 1 sugar alcohol, 1 sugar acid, and 10 from other acid, alcohol, or sugar chemical families. Subsequently, the metabolites with the highest VIP scores were selected for pathway analysis to observe impacted pathways during the gluconic acid metabolism. The pathway graph considers *p*-values and pathway impact to visually display those pathways that are highlighted. Those pathways which had high -log(p) and impact values were glycerolipid metabolism, glycine-serine-threonine metabolism, glyoxylate-dicarboxylate metabolism, and citrate cycle (TCA cycle or tricarboxylic acid cycle) (Figure 3). Focus is placed on these pathways and metabolites, which are visually represented as a metabolomic map (Figure 4).

Other pathways which linked with detected metabolites were glutathione metabolism, methane metabolism, aminoacyl-tRNA biosynthesis, pentose phosphate pathway, cyanoamino acid metabolism, thiamine metabolism, porphyrin and chlorophyll metabolism, sulfur metabolism, steroid biosynthesis, amino sugar and nucleotide sugar metabolism, starch and sucrose metabolism, alanine, aspartate and glutamate metabolism, biosynthesis of unsaturated fatty acids, arginine biosynthesis, pyrimidine metabolism, nitrogen metabolism, amino sugar and nucleotide sugar metabolism, tyrosine metabolism, propanoate metabolism, butanoate metabolism, nicotinate and nicotinamide metabolism, fructose and mannose metabolism, purine metabolism, and galactose metabolism (Supplementary Table S1).

**Figure 5:**
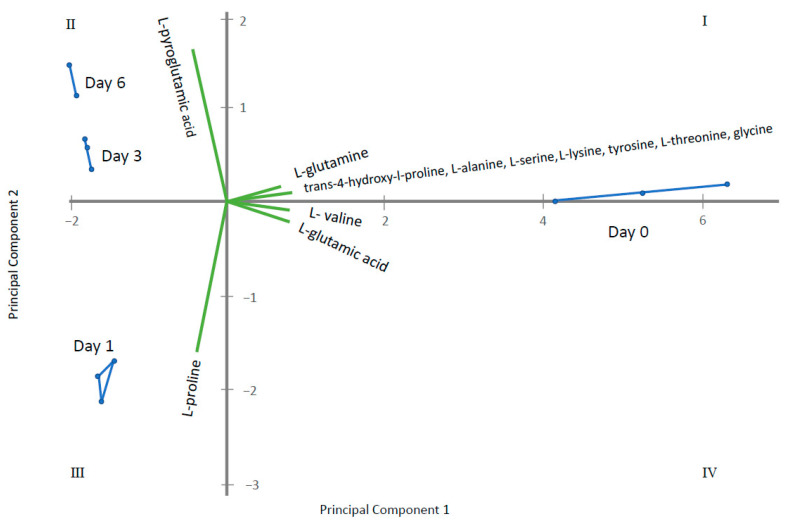
Principal Component Analysis (PCA) for amino acids where Principal Component 1 displays 85% of the variability and Principal Component 2—12.6%. The green vectors represent each metabolite, whose projection of the vertex on each axis represents its contribution to each PC. The blue points are each sampling time replicate, connected by lines to represent the PC scores of the samples analyzed.

**Figure 6:**
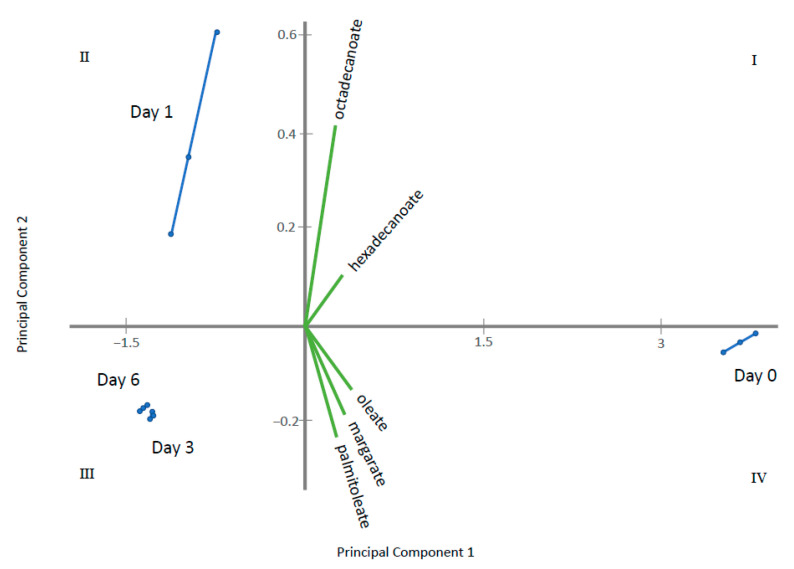
Principal Component Analysis (PCA) for fatty acids where Principal Component 1 displays 98.4% of the variability and Principal Component 1 for 1.4%. The green vectors represent each metabolite, whose projection of the vertex on each axis represents its contribution to each PC. The blue points are each sampling time replicate, connected by lines to represent the PC scores of the samples analyzed.

**Figure 7:**
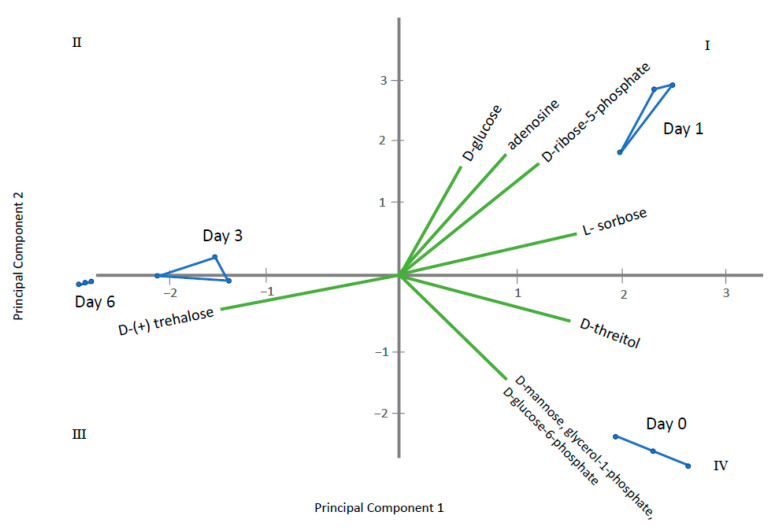
Principal Component Analysis (PCA) for sugars where Principal Component 1 displays 52.6% of the variability and Principal Component 2—37%. The green vectors represent each metabolite, whose projection of the vertex on each axis represents its contribution to each PC. The blue points are each sampling time replicate, connected by lines to represent the PC scores of the samples analyzed.

**Figure 8:**
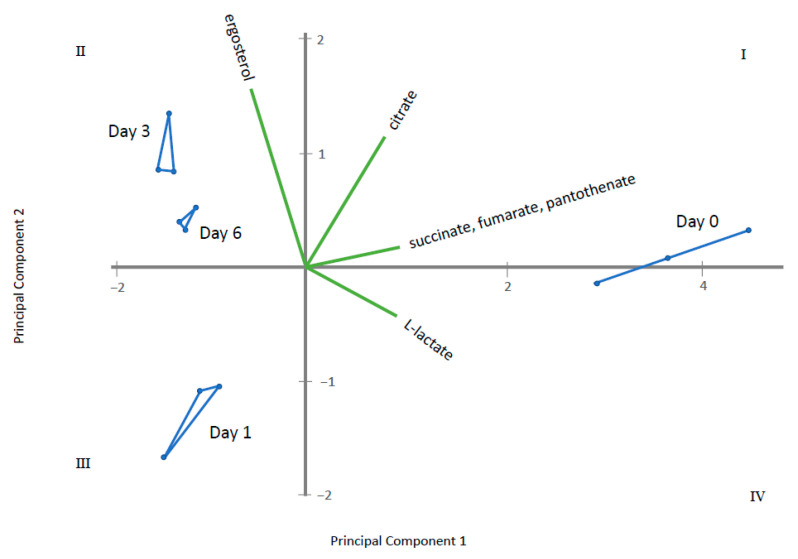
Principal Component Analysis (PCA) for other acids and other alcohols detected where Principal Component 1 displays 82.3% of the variability and Principal Component 2 16.4%. The green vectors represent each metabolite, whose projection of the vertex on each axis represents its contribution to each PC. The blue points are each sampling time replicate, connected by lines to represent the PC scores of the samples analyzed.

Through ANOVA and the Kruskal–Wallis test, homogeneous groups were established for metabolites displaying significant differences among the sampling times (Supplementary Table S2). Those having two or more homogeneous groups (*p* ≤ 0.05) were subjected to a Principal Component Analysis (PCA). In this way, four PCAs were obtained, corresponding to the four groups of metabolites previously established: amino acids, fatty acids, sugars and polyols and other acids and alcohols. The results obtained for each PCA are plotted in Figure 5, Figure 6, Figure 7 and Figure 8 that shows the contribution of each metabolite to the PCs and the sample scores for PC1 and PC2. In these biplots, each metabolite is represented by one vector, whose projection of the vertex on each axis represents its contribution to each PC. The points represent the PC scores of the samples analyzed.

Figure 5 displays amino acids where PC1 represents 85% of the variability and PC2 12.6%. l-lysine, l-serine, l-glutamine, hydroxyproline, glycine, l-valine, l-alanine, l-threonine tyrosine and l-glutamate are the most important contributors, with positive projections over PC1. By contrast, l-proline and l-pyroglutamic acid are important contributors to PC2. PC1 differentiate among control samples (at 0 days) and samples where gluconic acid consumption was observed. Figure 6 displays long chain fatty acids and PC1 represents 98.4% of the variability while PC2 is 1.4%. All fatty acids had positive projections over PC1, which show positive values for control sample and negative for the remaining samples. PC2 have positive values for samples taken at day 1 and negative for the remaining. This PC2 is mainly affected by C18 and C14 saturated fatty acids. Figure 7 represents different sugar metabolites detected where PC1 displays 52.6% of the variability and PC2 the 37.0%. d-glucose, adenosine, d-ribose-5-phosphate and l-sorbose have positive projections over PC1 and PC2 and the samples corresponding to 0 and 1 days have positive PC1 scores. Erythritol, d-glucose-6-phosphate, d-mannose, and glycerol-1-phosphate are projected in quadrant IV and closest to T0. Three other compounds, glycerol, fructose, and d-trehalose have negative projections over PC1 and near to zero value for PC2. Samples corresponding to 3 and 6 days show values near to zero for PC2 and negative values for PC1. The last PCA was obtained using the other acids and other alcohols as variables and PC1 shows 82.3% and PC2 of 16.4% of the total variance (Figure 8). Most metabolites lie in quadrant I: citrate, pantothenate, succinate and fumarate; while l-lactate lies in quadrant IV, contributing with positive values to the PC1. Ergosterol in quadrant II contributes to the PC2. The PC1 projects the sample at day 0 to the most positive values, the remaining samples have negative values and PC2 differentiate among samples taken at day 1, 3 and 6.

## 3. Discussion

Flor yeast differs from other strains of *S. cerevisiae* mainly for their ability to form a biofilm (“flor”) on the wine surface, allowing growth in a medium rich in dissolved oxygen, and development of aerobic metabolism [18]. This metabolism allows flor yeasts to be able to consume ethanol, non-fermentative carbon sources such as glycerol, acetic acid, ethyl acetate, amino acids (i.e., proline) and organic acids [2,19,20,21]. In this study, the focus is placed on gluconic acid consumption, which is favored by a proton symport system, similar to that of glycerol [6]. As visualized in Figure 4, once gluconic acid enters the cell, it is metabolized by the pentose phosphate pathway then to glycolysis, where hexoses are oxidized to generate ATP, reducing power and pyruvic acid. Glycolysis operates as an amphibolic pathway, acting both in forward and reverse directions in order to fulfill the lack of precursor metabolites or energy within the cell [22].

Branching from glycolysis, four other pathways are highlighted from the metabolism of gluconic acid as a sole carbon source: the TCA cycle, glyoxylate-dicarboxylate metabolism, glycine-serine-threonine metabolism, and glycerolipid metabolism (Figure 3, 4). The TCA cycle, along with the pentose phosphate pathway and glycolysis, make up the major pathways of central metabolism. This pathway is essential to reduce acetyl-CoA and to generate the reducing equivalents needed to produce ATP [23]. The TCA cycle includes intermediates that are an important source of carbon for amino acid synthesis and other biological compounds, giving the pathway a biosynthetic function. Although many of the TCA cycle enzymes are not utilized for yeast growth on fermentable carbon sources, they are significant for growth on non-fermentable carbon sources. Like the TCA cycle, the glyoxylate cycle begins with the same steps of converting oxaloacetate and acetyl-CoA to the six-carbon molecule isocitrate. However, rather than utilizing the acetyl units as the TCA cycle does, the glyoxylate cycle results in a net condensation of 4-carbon precursors. These two pathways allow *S. cerevisiae* to consume C2 carbon sources and fatty acids as the only sources of carbon [24,25,26,27]. Further, Moreno-Garcia et.al. [4] reported a high level of synthesis of enzymes involved in these pathways when growing flor yeasts under ethanol as the sole carbon source. In this study, the TCA cycle and glyoxylate-dicarboxylate metabolism appear more active at day 6 of incubation (Figure 2).

Glycine, serine, threonine metabolism involves the biosynthesis and catabolism of the proteinogenic amino acids necessary for the synthesis of proteins. There exist several pathways of these amino acids, starting with threonine which can be cleaved by threonine aldolase to form glycine and acetaldehyde [28,29]. The increase in acetaldehyde in sherry wines by flor yeast gluconic acid consumption has been reported by Peinado et al. 2004 [20]. Glycine can also be formed from glyoxylate by alanine glyoxylate aminotransferase (Agx1p). From here, glycine can be converted to serine by hydroxymethyltransferases Shm1p and Shm2p, a reaction which is reversible and interchangeable [30,31]. The concentrations of these metabolites were all low by day 6 (Figure 3) signifying that metabolism on gluconic acid induces the utilization of these amino acids or they were excreted out of the cell.

Lastly, glycerolipid metabolism is activated and driven mostly by the accumulation of glycerol towards day 6 of incubation. Glycerolipids (e.g., phospholipids and triacylglycerol) in yeasts have functions such as membrane trafficking, membrane structure, cell signaling and anchoring proteins to membranes [32]. Some of these functions are of relevance when the flor yeasts are in a condition depleted of nitrogen and non-fermentable carbon sources [33].

Apart from highlighted pathways, metabolites that presented high VIP scores and accumulation at day 3 or day 6 of incubation were l-pyroglutamic acid (5-oxoproline), ergosterol and fructose. l-glutimate or pyroglutamic acid is an amino acid that is an intermediate of glutathione biosynthesis and catabolism. Pyroglutamic acid can be directly converted to glutamic acid, an important nitrogen source for yeasts [34]. Ergosterol is a major constituent of fungal plasma membrane and concentrate with sphingolipids to form lipid rafts, which are specialized membrane microdomains that are responsible for cellular processes such as: organization of membrane lipids and proteins and regulating signaling cascades. The lipid rafts function as platforms to bring specific proteins to the cell surface as well as maintain cell polarity [35]. Additionally, an increase in ergosterol content in yeasts is tied to ethanol tolerance [36,37].

Another metabolite that was accumulated by day 6 of incubation was fructose. and closest to day 3 and day 6 in the PCA. Fructose is a monosaccharide that is a fermentable carbon source of the yeast. Although it is normally consumed, yeasts are able to synthesize it via several different origins, these include: d-sorbitol, sucrose-6-phosphate, sucrose and d-mannitol. Inside the cell, fructose is phosphorylated by the hexokinases, Hxk1p and Hxk2p, to fructose-6-phosphate. The expression of *HXK1* happens when there are non-fermentable carbon sources, such as gluconic acid or galactose [38,39,40]. Fructose-6-phosphate is well known as an intermediate of glycolysis, but also is used to form UDP-*N*-acetyl-d-glucosamine (UDP-GlcNAc), which is utilized for the biosynthesis of chitin, a major component that make up the structure of fungal cell walls. Conditional depletion of enzymes involved in the synthesis of UDP-GlcNAc can result in increased cell lysis and abnormal cell morphology, making UDP-GlcNAc essential for *S. cerevisiae* [40,41,42,43]. It is well known that flor yeasts change the structure and composition of the cell wall depending on the carbon source available in the medium [1,2] so we believe that gluconic acid may have an impact on the cell wall structure as well.

## 4. Materials and Methods

### 4.1. Microorganisms and Culture Conditions

*Saccharomyces cerevisiae* G1 (ATCC MYA-2451™, Cordoba, Spain) was utilized for this study. Yeast cells were cultured on YPD-agar (1% yeast extract, 2% peptone, 2% glucose, and 2% agar) and pre-inoculated in 50 mL of YPG medium (1% yeast extract, 2% peptone, 3% glycerol) in 100 mL flasks at 175 rpm and 28 °C for three days. YPG rather than YPD was selected to pre-adapt cells because gluconic acid consumption was faster when cells were pre-cultured in glycerol as a carbon source [15]. After finishing the pre-inoculation period, a high population of yeast cells, 4 × 10^6^ yeast cells/mL, was inoculated in 150 mL of gluconic acid medium in 250 mL flasks. The gluconic acid medium consists of yeast nitrogen base medium without amino acids (Difco), 5 g/L gluconic acid as the only carbon source, and buffered to pH 7 with 0.04 M Na_2_HPO_4_ and 0.03 M KH_2_PO_4_, which is optimum for gluconic acid consumption [6]. The flasks were shaken at 175 rpm, for 6 days in 28 °C. During this incubation period, samples were analyzed at day 0 (used as control sampling time), 1, 3, and 6 for the following parameters: consumption of gluconic acid, yeast cell viability, and identification/quantification of endo-metabolites. Contamination tests were carried out in each sample to ensure no other microorganism agents of gluconic consumption happened other than the flor yeast. Each sample was performed in triplicate.

### 4.2. Gluconic Acid Consumption

The concentration of gluconic acid in the medium was measured to assess its depletion. First, the complete medium was filtered with a Millipore filtration system using a 0.22 μm pore membrane, from Merck (Darmstadt, Germany) to achieve a liquid without yeast cells. This was subjected to analysis of gluconic acid by an enzymatic kit from Roche (Basel, Switzerland) and following the provider instructions. The absorbance read at 340 nm in a Beckman DU-640 UV spectrophotometer from Beckman Coulter Inc. (Brea, CA, USA), is directly proportional to the concentration of this acid.

### 4.3. Yeast Cell Viability

The viability of cells at each time point was analyzed by plating samples on YPD plates after serial dilutions and counting the number of single colonies after incubation at 28 °C for 3 days.

### 4.4. Endo-Metabolite Identification and Quantification

To analyze the metabolites within the yeast cells at each sampling time, 5 × 10^6^ cells were taken and pelleted by centrifugation at 5000 rpm (2236× *g* RCF), 22 °C for 10 min (Thermo Scientific TX-400 Rotor, Waltham, MA, USA) and immediately quenched to preserve metabolites. Quenching was done by mixing the pellet with 100% cold methanol at 1:1 ratio, vortexed, and centrifuged at 14,000 rpm (17,530× *g* RCF), 4 °C for 30 s. The pellet was then resuspended in 1 mL 50% cold methanol and centrifuged at 12,000 rpm (12,879× *g* RCF) at 4 °C for 2 min and then dried utilizing Eppendorf concentrator 5301 for 1.25 h until samples had a constant weight. Once dry, the pellets were stored at −80 °C. Next, metabolites were extracted following the protocol outlined in [44] in the following way: the dry pellets were combined with 1.5 mL of 75% boiling ethanol, 100 mg of glass beads and 15 μL of 3300 μM quinic acid (internal standard) and vortexed for 2 min in 30 s increments. The supernatant was separated into a new tube and heated on a hot plate for 3 min at 95 °C and subsequently cooled for 10 min at −20 °C. Immediately after, samples were placed in a Labconco Centrivap concentrator at 30 °C until all liquid was evaporated.

Derivatization of the extracted samples was achieved by adding 10 μL of 40 mg/mL O-methylhydroxylamine hydrochloride (CAS 593-56-6) in pyridine (CAS 110-86-1) and vortexing for 30 s and then incubated at 30 °C, 175 rpm for 90 min. After, 90 μL of *N*-methylsilytrifluoroacetamide (CAS 24589-78-4) with 1% trimethylchlorosilane (CAS 25561-30-2) was added and vortexed for 30 s followed by another incubation period of 37 °C, 175 rpm for 30 min.

Detection and analysis of endo-metabolites was performed using the analytical platform Gas Chromatography-Mass Spectromety Detector (GC-MSD) with GC 7890A and MSD 5975C instrument from Agilent Technologies Series Autosampler (Palo Alto, CA, USA) following the method of [44]. The software used in conjunction was Chemstation (Agilent Technologies, Santa Clara, CA, USA). The 7890A GC instrument had a 122-5532G column that was 30 m length × 250 μm diameter × 0.25 μm film (Agilent Technologies, Santa Clara, CA, USA). The derivatized samples were placed in GC-MSD vials with 2 μL of fatty acid methyl ester markers (FAME). Sample injection volume was set to 2 μL with an inlet heater temperature of 250 °C, 9.001 psi pressure and a total flow of 14.75 mL/min. Helium flow was at 1.0682 mL/min. Initial temperature was 60 °C hold for 1 min with a ramp rate at 10 °C/min to 325 °C and hold for 10 min. The MSD instrument was used for 5.90 min for solvent delay and 2047V EMV Mode. The Scanning Mass Range was 50 to 600 amu.

Each sampling time was measured in three replicates and each replicate identified by using FiehnLib [45], NIST08 and Wiley7 libraries. Only those metabolites that had a Quality value over 70 were selected and quantified as equivalents of IS μmol/g since certain reference standards were not available. Semi-quantitative values were computed by multiplying each relative area of the compound (total area of the compound/total area of the IS) by the IS response factor (IS μmol/IS total area) and dividing by the mass of the sample corresponding to the mass of yeasts taken to quench at each sampling time.

### 4.5. Statistics

Statistical treatment of data was done by the use of Metaboanalyst 4.0 [46] from Wishart Research Group (Alberta, Canada) to create the VIP (Variable Importance in Projection) score chart with heatmap and perform enrichment pathway analysis. Supervised recognition techniques such as partial least square discriminant analysis (PLS-DA) were also applied to study the metabolite discriminant power and to select potential metabolite markers through the VIP values. The VIP is “the weighted sum of squares of the PLS loadings taking into account the amount of explained Y-variation, in each dimension” and the *p*-value is calculated as “the proportion of the times that class separation based on randomly labeled sample is at least as good as the one based on the original data (one-sided p value)” (Metaboanalyst). To establish significant differences of each sample among the four different samplings times, homogenous was generated by a statistical software package, Statgraphics Plus v. 2 from STSC, Inc. (Rockville, MD, USA) via ANOVA and the Kruskal–Wallis test. Principal Component Analysis plots (PCA) were created using the software PAST from Palaeontological Association (Oslo, Norway) [47]. All results reported or shown are the average of three replicas in each condition.

## 5. Conclusions

As a summary, the presence and use of gluconic acid as a sole carbon source for the flor yeast strain contributed to impact on the following pathways: TCA cycle, glyoxylate-dicarboxylate metabolism, glycine-serine-threonine metabolism, and glycerolipid metabolism. Additionally, pyroglutamic acid, ergosterol and fructose accumulated more towards the latter half of the incubation period (day 3 and 6) possibly to synthesize nitrogen storage molecules, restructure the plasma membrane and form UDP-GlcNAc. The accumulation of these metabolites points to the possibility of yeast cells preparing and storing necessary carbon and nitrogen precursors to subsequently use them at a later stage to prolong viability. At the same time, the yeast cells may be physically changing structural components (i.e., cell wall) to become more robust. These changes may especially be highlighted because of the yeast strain used in this study, which is known to be highly tolerant to stress conditions. Among other *S. cerevisiae* strains, flor yeast strains dominate after alcoholic fermentation finishes, in carbon lacking and nutrient poor environments, because it can produce flocculation proteins on its cell walls for biofilm formation [1,2]. Thus, the metabolomic changes highlighted in this work may also be influenced by mechanisms by which the flor yeast strain responds to stress conditions such as the lack of fermentable carbon sources and nutrient limited conditions.

This work focused on those metabolites that highlighted the most impacted pathways and VIP scores, however, other metabolites that were detected but not discussed in full could form a valuable basis of knowledge for future studies with a final goal of gluconic acid consumption improvement by yeast strain engineering or redesigning preculture conditions. Further, enzyme activities and metabolic flux analysis can be future work that confirms these changes in intracellular metabolite concentration. The results presented here may contribute to the enhancement of some *S. cerevisiae* strains and their application as bioabsorption agents of harmful metabolites for others microorganism or biotechnological process.

## Data Availability

The data presented in this study are available in Supplementary Materials.

## Supplementary Materials

The following are available online at https://www.mdpi.com/2218-1989/11/3/150/s1, Table S1: Pathway match status of the top 15 endometabolites with highest VIP scores. Match status (number of metabolites in given data sample/total number of metabolites in the pathway), *p* value, −log(*p*), Holm p value, false discovery rate (FDR) of all generated pathways, Table S2: Average concentration (µmol/g) and standard deviations (SD) of endomentabolites detected in all conditions. Different letters (A–C) indicate homogeneous groups (HG) statistically significantly differing in values of concentration of the endometabolite among the conditions (*p* < 0.05, F-test).
